# Prevalence and factors associated with the use of primary headache diagnostic criteria by chiropractors

**DOI:** 10.1186/s12998-019-0254-y

**Published:** 2019-08-06

**Authors:** Craig Moore, Andrew Leaver, David Sibbritt, Jon Adams

**Affiliations:** 1 Faculty of Health, University of Technology Sydney, Level 8, Building 10, 235-253 Jones Street, Ultimo NSW, Sydney, 2007 Australia; 2 Faculty of Health Science, University of Sydney, Sydney, Australia

**Keywords:** Chiropractic, Headache diagnosis, Migraine, Tension headache, Cluster headache, Manual therapy, Practice-based research network (PBRN)

## Abstract

**Background:**

The diagnosis of primary headaches assists health care providers in their decision-making regarding patient treatment, co-management and further evaluation. Chiropractors are popular health care providers for those with primary headaches. The aim of this study is to examine the clinical management factors associated with chiropractors who report the use of primary headache diagnostic criteria.

**Methods:**

A cross-sectional survey was distributed between August and November 2016 to a random sample of Australian chiropractors who are members of a practice-based research network (*n* = 1050) who had reported ‘often’ providing treatment for patients with headache disorders to report on practitioner approaches to headache diagnosis, management, outcome measures and multidisciplinary collaboration. Multiple logistic regression was conducted to assess the factors that are associated with chiropractors who report using International Classification of Headache Disorders (ICHD) primary headache diagnostic criteria.

**Results:**

With a response rate of 36% (*n* = 381), the majority of chiropractor’s report utilising ICHD primary headache diagnostic criteria (84.6%). The factors associated with chiropractors who use ICHD primary headache diagnostic criteria resulting from the regression analysis include a belief that the use of ICHD primary headache criteria influences the management of patients with primary headaches (OR = 7.86; 95%CI: 3.15, 19.60); the use of soft tissue therapies to the neck/shoulders for tension headache management (OR = 4.33; 95%CI: 1.67, 11.19); a belief that primary headache diagnostic criteria are distinct for the diagnosis of primary headaches (OR = 3.64; 95%CI: 1.58, 8.39); the use of headache diaries (OR = 3.52; 95%CI: 1.41, 8.77); the use of ICHD criteria improves decision-making regarding primary headache patient referral/co-management (OR = 2.35; 95%CI: 1.01, 5.47); referral to investigate a headache red-flag (OR = 2.67; 95%CI: 1.02, 6.96) and not referring headache patients to assist headache prevention (OR = 0.16; 95%CI: 0.03, 0.80).

**Conclusion:**

Four out of five chiropractors managing headache are engaged in the use of primary headache diagnostic criteria. This practice is likely to influence practitioner clinical decision-making around headache patient management including their co-management with other health care providers. These findings call for a closer assessment of headache characteristics of chiropractic patient populations and for further enquiry to explore the role of chiropractors within interdisciplinary primary headache management.

## Background

The global adult prevalence of tension-type headache and migraine is reported to be approximately 40 and 10%, respectively [1–3]. These headaches constitute a substantial burden on the personal health and productivity of sufferers [4, 5] and cause a significant drain on healthcare resources [6, 7]. While those with chronic tension headache can sometimes report greater headache pain than those with migraine [8], migraine is one of the top 10 causes of years lived with disability [9] and the third leading cause of disability for those under the age of fifty [10].

Significant challenges remain regarding the management of headache patients. Headache patients are often poorly or under diagnosed [11], under treated [12, 13] or can fail to receive effective interdisciplinary management [14, 15]. Such challenges have led international headache organisations [16–18] and headache researchers [19, 20] to call for more effective health care service delivery for this significant patient population. While general practitioners (GPs) are typically the first point of contact for those with primary headaches [7, 21], sufferers can enter the healthcare system via a range of health care providers [22–24]. The use of chiropractors for headache management is likely most often for primary headaches, with studies reporting substantial use in the North America [25, 26], Australia [27] and parts of Europe [24, 28]. Despite the substantial use of chiropractors by those with primary headaches, little is known about how these practitioners manage this patient population. Such information can improve our understanding of headache-related health care delivery services and the role of these providers within the wider landscape of headache patient management.

The 3rd edition of International Classification of Headache Disorders (ICHD) outlines the current criteria utilised for headache diagnosis [29]. Headache diagnosis is a key determinant that will influence practitioner decision-making around headache patient care. While a recent study reported the high use of headache diagnosis by chiropractors [30], there is little information regarding how primary headache diagnosis influences the clinical management of headaches by these providers. In direct response, the aim of this study was to draw upon a national sample of chiropractors to identify the headache patient management factors associated with those practitioners who utilise.

## Methods

The data analysed in this study was drawn from a questionnaire distributed to members (chiropractors) of a national practice-based research network (PBRN) titled the Australian Chiropractic Research Network (ACORN) project [31]. This study was approved by the Human Research Ethics Committee at the University of Technology Sydney (Approval number ETH16–0639).

### Recruitment and sample

Detailed information about the ACORN PBRN recruitment and data base has been previously reported [31, 32], but briefly, ACORN recruitment was conducted via an invitation pack that included a baseline questionnaire disseminated between March and June 2015 to all registered Australian chiropractors. Invitation pack distribution was via email (with an embedded link to online questionnaire), postal distribution (hard copy questionnaire), regional chiropractic conferences (hard copy questionnaire) and the official ACORN website (with an embedded link to the online questionnaire). Forty-three percent (*n* = 1680) of all registered Australian chiropractors joined the ACORN network database. The socio-demographic profile of the ACORN database is representative of the wider chiropractic profession across Australia in terms of gender, age and practice location [32].

Participants for this PBRN sub-study were randomly selected from the ACORN practitioners who had reported that they ‘often’ provided treatment for patients with headache disorders in the ACORN PBRN invitation pack questionnaire. Participants were asked to complete a 31-item cross-sectional online survey between August and November 2016. An embedded link to the questionnaire was emailed to chiropractors. Three further reminders to complete the survey were sent out during the recruitment period. Participation in the survey was further promoted within routine email newsletters sent out by the Australian Chiropractors Association during that period.

### Questionnaire

The introduction to the questionnaire explained the purpose, contents and approximate duration of the study and that respondent information was anonymous and survey completion voluntary. No incentives were offered to participate, and consent was implied by completing the survey. Questionnaire items specifically developed for this study aimed to examine chiropractic headache management across several clinical themes considered important to frontline headache management practice. With no validated instruments available, the key themes adopted for our study questionnaire were developed after consideration of past surveys examining the management of headache patients in primary care settings [11, 33, 34]. The survey collected information on practitioner characteristics, including gender, place of education, practice location and years in practice. Prevalence of headache in chiropractic practice was based on self-report on patient consultations over the previous two weeks. The use of formal diagnostic criteria for headaches was based on International Classification of Headache Disorders (ICHD-3 Beta) criteria [35]. The survey design included descriptions of primary headache criteria for migraine, tension-type headache and cluster headache and for secondary headache criteria for cervicogenic and medication overuse headache. The use of headache treatment outcome instruments was based on the use of the Headache Disability Index (HDI) [36], Migraine Disability Index (MIDAS) [37] and standard patient headache diaries [38]. Patient management included questions on collaboration with other healthcare providers associated with headache management (sending and receiving) and questions on the basis for patient referral. The questions on headache management provided a list of therapeutic approaches for headache including patient education on headache triggers, physical therapies and manual therapies utilised for headache (e.g. spinal manipulation, mobilisation, massage therapy).

The primary headache management questions included in the questionnaire were based upon primary headaches previously reported as most often treated by chiropractors [24, 39, 40] and after consultation with 10 practicing Australian chiropractors during survey pilot testing. The pilot testing findings were discussed between all members of the research team to assist decisions about survey duration and the selection of the survey themes and item options. This included the selection of headache treatment outcome measures, where we considered practitioner familiarity and understanding of the nature and purpose of select outcome instruments as well as treatment terminology and their views on relevance to headache management. All questionnaire items were either reported as ratings on a 4-point or 5-point Likert scale or as dichotomous (yes/no).

### Statistical analyses

Summary statistics were presented by number (percentage), mean (SD) as appropriate. In-order to test the differences in continuous and categorical variables by group, we have used Student’s t-test and chi-square test or Fishers exact test respectively (Table 1). Bivariate comparison of clinical management characteristics, headache referral characteristics, importance of headache treatment outcomes and headache management characteristics were made between chiropractors who indicated the use ICHD headache classification criteria for the diagnosis of primary headaches (i.e. yes/no) using chi-square/Fishers exact test as appropriate (Tables 2, 3, 4, 5).

**Table 1:** Comparison of survey population with ACORN membership based on demographic characteristics

Characteristic	Survey Population	ACORN Database	*p*-value
Gender (%)			
Male	64	63	*p* = 0.379
Female	36	37	
Place of Practice (%)			*p* = 0.916
New South Wales	35.1	34	
Victoria	23.2	25	
Queensland	15.2	15	
Western Australia	14.7	13	
South Australia	8.5	9	
Australian Capital Territory	1.6	2	
Tasmania	0.9	1	
Northern Territory	0.5	1	
Place of Education (%)			
New South Wales	38.6	N. A	
Victoria	35.6	N. A	
Queensland	0.8	N. A	
Western Australia	9.7	N. A	
Other	15.3	N. A	
Years in Practice (mean ± sd)	18.1 ± 10.9	15.6 ± 11.2	*p* = 0.003

*N. A* denotes comparative data not available for place of education

**Table 2:** Clinical management characteristics across the use of ICHD primary headache diagnostic criteria

Chiropractic headache classification/assessment		Used diagnostic criteria for primary headache types		
		No (*n* = 61)	Yes (*n* = 334)	*p*-value
Headache classification criteria (views)		%	%	
ICHD primary headache diagnostic criteria are distinct for the diagnosis of primary headaches	Strongly disagree	3	1	< 0.001
	Disagree	10	4	
	Neutral	34	10	
	Agree	46	66	
	Strongly agree	7	19	
ICHD primary headache diagnostic criteria are easy to follow	Strongly disagree	5	1	< 0.001
	Disagree	5	2	
	Neutral	23	8	
	Agree	62	67	
	Strongly agree	5	22	
Patients with primary headache easily fit into ICHD primary headache diagnostic criteria	Strongly disagree	6	1	< 0.001
	Disagree	43	17	
	Neutral	25	29	
	Agree	23	46	
	Strongly agree	3	5	
ICHD primary headache diagnostic criteria influences headache management	Strongly disagree	13	2	< 0.001
	Disagree	41	9	
	Neutral	28	17	
	Agree	18	58	
	Strongly agree	0	14	
ICHD primary headache diagnostic criteria helps communication with other healthcare professionals	Strongly disagree	5	0	< 0.001
	Disagree	13	1	
	Neutral	36	14	
	Agree	41	60	
	Strongly agree	5	25	
ICHD primary headache diagnostic criteria improves decision-making about patient referral or co-management	Strongly disagree	10	1	< 0.001
	Disagree	29	4	
	Neutral	36	17	
	Agree	23	58	
	Strongly agree	2	20	
Headache outcome criteria				
Use of Migraine Disability Assessment Test (MIDAS)	Never	97	69	< 0.001
	Rarely	3	27	
	Often	0	4	
	Every new headache patient	0	1	
Use of Headache Disability Inventory (HDI)	Never	91	60	< 0.001
	Rarely	9	24	
	Often	0	12	
	Every new headache patient	0	3	
Use of Headache Diary	Never	43	18	< 0.001
	Rarely	40	37	
	Often	14	42	
	Every new headache patient	3	4

**Table 3:** Headache referral characteristics across the use of ICHD primary headache diagnostic criteria

Chiropractic headache referral		Used diagnostic criteria for primary headache types		
		No (*n* = 61)	Yes (*n* = 334)	p-value
Headache referral (receiving)		%	%	
Headache referral from GP	Never	42	27	< 0.001
	Rarely	46	40	
	Sometimes	7	29	
	Often	5	4	
Headache referral from medical specialist (neurologist, rheumatologist, orthopaedic, psychiatrist)	Never	89	77	< 0.001
	Rarely	9	19	
	Sometimes	2	4	
	Often	0	1	
Headache referral from Psychologist	Never	89	68	< 0.001
	Rarely	9	19	
	Sometimes	2	11	
	Often	0	2	
Headache referral from Dentist	Never	67	40	0.002
	Rarely	21	33	
	Sometimes	9	23	
	Often	4	5	
Headache referral from Physiotherapist	Never	74	67	0.684
	Rarely	19	21	
	Sometimes	7	10	
	Often	0	2	
Headache referral from Osteopath	Never	89	78	0.154
	Rarely	11	16	
	Sometimes	0	5	
	Often	0	1	
Headache referral from CAM practitioners (inc. acupuncturist, herbalist, naturopath, massage therapist, counsellor)	Never	28	11	< 0.001
	Rarely	28	19	
	Sometimes	35	47	
	Often	9	23	
Headache referral (sending)				
Headache referral to GP	Never	19	7	< 0.001
	Rarely	46	29	
	Sometimes	33	55	
	Often	2	9	
Headache referral to medical specialist (neurologist, rheumatologist, orthopaedic, psychiatrist) via GP	Never	25	17	< 0.001
	Rarely	58	36	
	Sometimes	18	42	
	Often	0	4	
Headache referral to Psychologist	Never	74	47	0.004
	Rarely	19	34	
	Sometimes	7	16	
	Often	0	2	
Headache referral to Dentist	Never	39	15	0.001
	Rarely	32	42	
	Sometimes	26	37	
	ften	4	5	
Headache referral to Physiotherapist	Never	72	54	0.106
	Rarely	21	31	
	Sometimes	7	13	
	Often	0	2	
Headache referral to Osteopath	Never	91	74	0.025
	Rarely	7	21	
	Sometimes	2	4	
	Often	0	0	
Headache referral to CAM practitioners (eg. acupuncturist, naturopath, massage therapist, counsellor)	Never	23	7	0.001
	Rarely	28	23	
	Sometimes	33	50	
	Often	16	20	
Headache referral (reasons)				
Headache referral to confirm headache diagnosis	Never	54	21	< 0.001
	Rarely	32	43	
	Sometimes	12	32	
	Often	2	4	
Headache referral to improve coping skills and headache disability management	Never	39	11	< 0.001
	Rarely	26	31	
	Sometimes	33	47	
	Often	2	10	
Headache referral to investigate headache red-flag	Never	0	1	< 0.001
	Rarely	42	12	
	Sometimes	42	51	
	Often	16	36	
Headache referral to provide pain relief for acute headache attacks	Never	26	10	0.004
	Rarely	33	29	
	Sometimes	33	48	
	Often	7	12	
Headache referral to help provide headache prevention	Never	37	17	0.004
	Rarely	33	35	
	Sometimes	25	39	
	Often	5	9

**Table 4:** Importance of headache treatment outcomes across the use of ICHD primary headache diagnostic criteria

Chiropractic headache management/treatment		Used diagnostic criteria for primary headache types		
		No (*n* = 61)	Yes (*n* = 334)	*p*-value
Importance of treatment outcomes		%	%	
Treatment aimed to prevent headache episodes	Very unimportant	11	5	0.317
	Somewhat unimportant	0	0	
	Neutral	0	1	
	Somewhat important	13	10	
	Very important	77	84	
Treatment aimed to improve recovery from episode of headaches	Very unimportant	9	4	0.043
	Somewhat unimportant	2	1	
	Neutral	0	1	
	Somewhat important	27	16	
	Very important	62	78	
Treatment aimed at pain relief during headache episode	Very unimportant	9	3	0.049
	Somewhat unimportant	0	1	
	Neutral	5	3	
	Somewhat important	41	32	
	Very important	45	61	
Treatment aimed to improve headache coping skills	Very unimportant	7	2	0.001
	Somewhat unimportant	11	3	
	Neutral	16	8	
	Somewhat important	32	35	
	Very important	34	52	
Treatment aimed to improve overall health and well-being	Very unimportant	9	4	0.411
	Somewhat unimportant	0	2	
	Neutral	2	3	
	Somewhat important	12	16	
	Very important	77	75

**Table 5:** Headache management characteristics across the use of ICHD primary headache diagnostic criteria

Chiropractic headache management		Used diagnostic criteria for primary headache types		
		No (*n* = 61)	Yes (*n* = 334)	*p*-value
Chiropractic headache management - migraine		%	%	
Manual adjusting/manipulation (including Diversified, Gonstead)	Never	7	5	0.751
	Rarely	14	12	
	Often	45	51	
	Almost every migraine patient	34	32	
Non-thrust spinal mobilisations	Never	18	9	0.001
	Rarely	37	19	
	Often	34	58	
	Almost every migraine patient	11	14	
Instrument adjusting	Never	14	9	0.407
	Rarely	18	18	
	Often	48	58	
	Almost every migraine patient	20	14	
Drop piece, Thompson or similar	Never	29	29	0.944
	Rarely	34	37	
	Often	22	28	
	Almost every migraine patient	5	6	
Massage, myofascial technique, stretching or trigger-points to neck/shoulder area	Never	9	2	< 0.001
	Rarely	25	9	
	Often	48	42	
	Almost every migraine patient	18	47	
Electro-physical therapies (TENS, ultrasound etc)	Never	89	77	0.236
	Rarely	9	15	
	Often	2	7	
	Almost every migraine patient	0	2	
Soft tissue or exercise therapy to temporomandibular region	Never	29	5	< 0.001
	Rarely	20	27	
	Often	41	54	
	Almost every migraine patient	11	14	
Prescriptive exercises for the neck/shoulder region	Never	14	2	< 0.001
	Rarely	20	14	
	Often	46	52	
	Almost every migraine patient	20	33	
Advice on stress management	Never	2	0	0.019
	Rarely	16	9	
	Often	59	51	
	Almost every migraine patient	23	41	
Advice on diet or fitness	Never	2	1	0.057
	Rarely	12	14	
	Often	64	49	
	Almost every migraine patient	21	37	
Advice on Headache triggers	Never	0	0	0.005
	Rarely	14	5	
	Often	52	45	
	Almost every migraine patient	34	50	
Chiropractic headache management - tension headache				
Manual adjusting/manipulation (including Diversified, Gonstead)	Never	9	4	0.291
	Rarely	11	7	
	Often	36	42	
	Almost every tension headache patient	45	47	
Non-thrust spinal mobilisations	Never	27	11	0.003
	Rarely	27	21	
	Often	38	52	
	Almost every tension headache patient	9	16	
Instrument adjusting	Never	14	11	0.810
	Rarely	20	18	
	Often	48	55	
	Almost every tension headache patient	18	16	
Drop piece, Thompson or similar	Never	32	35	0.662
	Rarely	25	27	
	Often	38	30	
	Almost every tension headache patient	5	8	
Massage, myofascial technique, stretching or trigger-points to neck/shoulder area	Never	5	3	< 0.001
	Rarely	27	6	
	Often	43	40	
	Almost every tension headache patient	25	51	
Electro-physical therapies (TENS, ultrasound etc)	Never	89	79	0.374
	Rarely	9	13	
	Often	2	6	
	Almost every tension headache patient	0	2	
Soft tissue or exercise therapy to temporo- mandibular region	Never	18	8	0.017
	Rarely	32	25	
	Often	43	50	
	Almost every tension headache patient	7	18	
Prescriptive exercises for the neck/shoulder region	Never	11	1	< 0.001
	Rarely	20	8	
	Often	45	45	
	Almost every tension headache patient	25	46	
Advice on stress management	Never	2	0	0.002
	Rarely	14	9	
	Often	59	42	
	Almost every tension headache patient	25	49	
Advice on diet or fitness	Never	2	2	0.480
	Rarely	11	11	
	Often	59	48	
	Almost every tension headache patient	29	39	
Headache triggers advice	Never	7	0	< 0.001
	Rarely	14	7	
	Often	46	47	
	Almost every tension headache patient	32	46	

Multiple logistic regression modelling was then performed to identify independent predictors associated with those chiropractors who use ICHD primary headache criteria (presented in Table 6). Questionnaire response items are dichotomized into “Strongly disagree/Disagree/Neutral” versus “Agree/Strongly agree” with bivariate associations of *p* < 0.2 included in the regression model. The independent survey variables were dichotomized after consideration of previous research [41, 42] and the distribution of the data. A backward stepwise procedure was chosen to determine the most parsimonious model that predicts those chiropractors who use primary headache diagnostic criteria. Statistical significance was set at *p* < 0.05. Odds ratios were reported with 95% confidence intervals. All statistical analyses were conducted using the statistical software Stata 13.1.

**Table 6:** Logistic regression analysis identifying associations with chiropractors who use ICHD primary headache diagnostic criteria

Factors		Odds Ratio	95% C.I.	*p*-value
ICHD primary headache diagnostic criteria influences headache management	Strongly Disagree/Disagree/Neutral	1.00		
	Agree/Strongly Agree	7.86	3.15, 19.60	< 0.001
ICHD primary headache diagnostic criteria improves decision-making about headache patient referral/co-management	Strongly Disagree/Disagree/Neutral	1.00		
	Agree/Strongly Agree	2.35	1.01, 5.47	0.046
Referral to investigate a headache red-flag	Never/Rarely/Sometimes	1.00		
	Often	2.67	1.02, 6.96	0.045
Referral to assist headache prevention	Never/Rarely/Sometimes	1.00		
	Often	0.16	0.03, 0.80	0.026
ICHD primary headache diagnostic criteria are distinct for the diagnosis of primary headaches	Strongly Disagree/Disagree/Neutral	1.00		
	Agree/Strongly Agree	3.64	1.58, 8.39	0.002
Massage, myofascial technique, stretching or trigger-points to neck/shoulder area for tension headache management	Never/rarely	1.00		
	Often/Almost every new patient with tension headache	4.33	1.67, 11.19	0.003
Headache diary	Never/Rarely	1.00		
	Often/Every new patient with headache	3.52	1.41, 8.77	0.007

## Results

A total of 1050 chiropractors were invited to participate of which 381 (36.2%) completed the questionnaire. As shown in Table 1, the sub-study sample of participants was compared to the wider ACORN sample and was shown to be similar across gender (*p* = 0.379), place of practice (*p* = 0.916) suggesting survey respondents are generally representative (non-significant *p* values) of the ACORN database participants while our sample was slightly more experienced than the ACORN database members for years in practice (*p* = 0.003). The majority of questionnaire respondents were male (64%) and the average number of years in practice was 18.1 (SD = 10.9) years. Most participants were educated in Australia including New South Wales (38.6%), Victoria (35.6%), Western Australia (9.7%) and Queensland (0.8%). Place of practice amongst the participants was greatest in New South Wales (35.1%), followed by Victoria (23.2%), Queensland (15.2%), Western Australia (14.7%), South Australia (8.5%), Australian Capital Territory (1.6%), Tasmania (0.9%) and Northern Territory (0.5%). Participant demographic characteristics were consistent with national chiropractic registration records [43].

### Factors associated with ICHD use for primary headaches

The majority of chiropractors reported utilising ICHD criteria for the diagnosis of primary headaches (84.6%). The clinical management characteristics of chiropractors who use or do not use the ICHD primary headache classification criteria are presented in Table 2. Chiropractors who use ICHD diagnostic criteria for primary headaches were more likely to believe that: ICHD criteria are distinct for the diagnoses of primary headache types; believe ICHD criteria are easy to follow; believe primary headaches easily fit into ICHD diagnostic criteria; believe ICHD criteria influences management of patients with primary headaches; ICHD criteria helps communication with other healthcare professionals; and improves decision-making about patient referral or co-management for those with primary headaches (all *p* < 0.001). In addition, those chiropractors who use primary headache diagnostic criteria were also more likely to use a Migraine Disability Assessment Test (MIDAS); the Headache Disability Inventory (HDI); and patient headache diaries (all *p* < 0.001).

Table 3 shows the referral characteristics of chiropractors who use or do not use the ICHD primary headache classification criteria. Chiropractors who used the ICHD primary headache diagnostic criteria were more likely to receive a headache referral from a general practitioner, medical specialist (including neurologist, rheumatologist, orthopaedic, psychiatrist), psychologist, CAM practitioners (including acupuncturist, herbalist, naturopath, massage therapist, counsellor) (all *p* < 0.001) and dentist (*p* = 0.002), compared to chiropractors who do not use the ICHD primary headache diagnostic criteria and less likely to receive headache referrals from a physiotherapist (*p* = 0.684) or osteopath (*p* = 0.154) although these associations were not statistically significant. Further, chiropractors who use the ICHD primary headache diagnostic criteria were also more likely to refer headache patients for further management to general practitioners (*p* < 0.001), medical specialists (*p* < 0.001), psychologist (*p* = 0.004), dentist (*p* = 0.001), and CAM practitioners (including acupuncturist, herbalist, naturopath, massage therapist, counsellor) (*p* = 0.001), compared to chiropractors do not use the ICHD primary headache diagnostic criteria and were less likely to refer a headache patient to a physiotherapist (*p* = 0.106) although these associations were not statistically significant. Chiropractors who use ICHD primary headache criteria were more likely to refer patients for reasons of confirming headache diagnosis (*p* < 0.001), improve coping skills (*p* < 0.001), investigate headache red-flags (*p* < 0.001), provide pain relief for acute headache attacks (*p* = 0.004) and to provide headache prevention (*p* = 0.004), compared to chiropractors do not use the ICHD primary headache diagnostic criteria.

The importance of headache treatment outcomes of chiropractors who use or do not use the ICHD primary headache classification criteria are presented in Table 4. Chiropractors who use ICHD primary headache criteria are more likely to aim their treatment toward improving the recovery from an episode of headaches i.e. postdromal headache period (*p* = 0.043), to provide pain relief during headache episode (*p* = 0.049) and improve headache related coping skills (*p* = 0.001) and less likely to aim treatment toward headache prevention (*p* = 0.317) or to improve headache patient overall health and well- being (*p* = 0.411) although these associations were not statistically significant.

Table 5 shows the approaches to primary headache management of chiropractors who use or do not use the ICHD primary headache classification criteria. For patients with migraine, chiropractors who use primary headache diagnostic criteria were also more likely to: provide non-thrust spinal mobilisations (*p* = 0.001); provide massage, myofascial technique, stretching or trigger-points to neck/shoulder area (*p* < 0.001); use soft tissue or exercise therapy to temporo-mandibular region (*p* < 0.001); prescribe exercises for the neck and shoulder region (*p* < 0.001); provide advice on stress management (*p* = 0.019) and headache triggers (*p* = 0.005), compared to chiropractors do not use the ICHD primary headache diagnostic criteria. They were less likely to provide manual manipulation (*p* = 0.751), instrument adjusting (*p* = 0.407), drop piece adjusting (0.944), electro-physical therapies (*p* = 0.236) and advice on diet or fitness (*p* = 0.057) although these associations were not statistically significant. For patients with tension headache, chiropractors who use primary headache diagnostic criteria were more likely to: provide non-thrust spinal mobilisations (*p* = 0.003); use massage, myofascial technique, stretching or trigger-points to neck/shoulder area (*p* < 0.001); use soft tissue or exercise therapy to temporomandibular region (*p* = 0.017); prescribe exercises for the neck/shoulder region (*p* < 0.001); provide advice on stress management (*p* = 0.002) and headache triggers (*p* < 0.001), compared to chiropractors do not use the ICHD primary headache diagnostic criteria. They were less likely to provide manual manipulation (*p* = 0.291), instrument adjusting (*p* = 0.810), drop piece adjusting (*p* = 0.662), electro-physical therapies (*p* = 0.374), and advice on diet and fitness (*p* = 0.480) although these associations were not statistically significant.

The results of the multiple logistic regression modelling used to identify the important independent factors associated with chiropractors who use ICHD primary headache diagnostic criteria compared to those chiropractors who do not use ICHD primary headache diagnostic criteria are presented in Table 6. These factors include a belief that: the use of ICHD primary headache criteria will influence their management of patients with primary headaches (OR = 7.86; 95%CI: 3.15, 19.6); improve decision-making about primary headache patient referral/co-management (OR = 2.35; 95%CI: 1.01, 5.47); and not referring headache patients to assist with headache prevention (OR = 0.16; 95%CI: 0.03, 0.80). Chiropractors who use ICHD criteria for the diagnosis of primary headaches are also associated with: believing ICHD criteria are distinct criteria for the diagnoses of primary headache types (OR = 3.64; 95%CI: 1.58, 8.39); ICHD primary headache diagnostic criteria influences the use of soft-tissue therapies to neck and shoulder region for tension headache (OR = 4.33; 95%CI: 1.67, 11.19); the use headache diaries as a headache outcome measure (OR = 3.52; 95%CI: 1.41, 8.77) and referral to investigate a headache red-flag (OR = 2.67; 95%CI: 1.02, 6.96).

## Discussion

This is the first study to provide detailed information on the patient management features associated with primary headache diagnosis by chiropractors. The majority of chiropractors in our study report utilising ICHD criteria for the diagnosis of primary headaches, a finding which may suggest that chiropractors are sometimes the first point of provider contact for patients seeking help for the management of primary headache disorders. There are a number of factors that can challenge health care providers delivering an accurate primary headache diagnosis. These include the co-occurrence of migraine with both cervicogenic headache [44] and tension-type headache [45], variations in headache characteristics found within headache types [46] and the high prevalence of co-occurring neck pain associated with common recurrent headaches [47, 48]. With misdiagnosis resulting in suboptimal headache patient management [49, 50], poor standards of headache diagnosis have raised concerns about the current level of headache education within primary health care curriculums [11, 49, 51]. Our study found almost half of those chiropractors engaged in primary headache diagnosis implement the use of patient headache diaries. The mixed use of headache diaries has been reported in other primary care settings [52]. While this practice is likely to improve diagnostic accuracy [38, 53], further research would be valuable in assessing the reliability of primary headache diagnosis as undertaken by chiropractors, information that can similarly inform chiropractic educational curriculums. Despite the high percentage of chiropractors self-reporting the utilisation of ICHD primary headache diagnostic criteria, uncertainty remains regarding how effectively chiropractors identify headache criteria in order to provide an accurate headache diagnosis.

Our study found several factors that were associated with chiropractors engaged in primary headache diagnosis. These chiropractors include a belief that the use of ICHD primary headache criteria influences their patient management. Previous studies have reported the use of manual therapies, exercise therapies and advice on headache triggers as common to chiropractic headache management [30, 54]. While advice on headache triggers is well recognised as an important aspect of primary headache management [55, 56], the effectiveness of manual and exercise therapies for the prevention of primary headaches requires further evaluation. To date, research evidence supports the role of manual and exercise therapies for the preventative treatment of tension headache [57, 58], while research supporting the role of these therapies for the prevention of migraine remains low quality and inconclusive [59, 60]. In contrast, around 10% of chiropractors who use primary headache diagnosis were not associated with ICHD primary headache diagnostic criteria influencing headache management. While this finding requires further investigation, it may be that providing a diagnosis of the patient’s headache type relates more to other motivations for a small number of practitioners. This could include to inform the headache patient or satisfy potential oversight from regulatory authorities. As such, more research is needed to examine how aspects of primary headache patient management are potentially improved through the use of primary headache diagnosis within chiropractic clinical settings.

Our analysis found several factors associated with chiropractors engaged in primary headache diagnosis that were related to specific aspects of practitioner decision-making regarding headache patient management. For example, our study found chiropractors engaged in the use of ICHD primary headache diagnostic criteria are more likely to believe doing so improves decision-making related to headache patient referral/co-management. The health care needs of primary headache sufferers can sometimes be multifactorial and multidisciplinary in nature, particularly for those who present with more complex and chronic headache conditions where a greater use of pharmaceutical, behavioural and physical approaches to patient care may be needed [61, 62]. Previous research has suggested that those with headaches seeking help from manual therapy providers are more likely have a higher rate of headache chronicity and disability than non-users [40]. As such, the belief that primary headache diagnosis improves decision-making about headache patient referral/co-management associated with chiropractors engaged in primary headache diagnosis may reflect practitioner awareness regarding the multidisciplinary health care needs of many primary headache patients within chiropractic patient populations [14, 63].

An unexpected finding from our results was that chiropractors engaged in primary headache diagnosis are less likely to undertake patient referral to assist with headache prevention. A recent Australian study showed chiropractors refer headache patients to both complementary health care providers (including acupuncturist, herbalist, naturopath, massage therapist, counsellor) and general practitioners [30]. For tension headache, preventative treatment guidelines advise non-drug management first be considered [64] and provide recommendations for behavioural treatments such as electromyography (EMG) biofeedback (level A), cognitive-behavioral therapy and relaxation training (level C), massage therapy (level C) and acupuncture (level C). In contrast, preventative treatment guidelines for migraine provide stronger recommendations for drug treatments (Level A) with additional recommendations also provided for several herbs and supplements such as butterbur (Level A), feverfew and magnesium (Level B) and coenzyme Q10 (level C) [65, 66]. Beyond headache diagnosis, provider referral to assist headache prevention requires careful consideration regarding a range of patient factors and circumstances including headache severity, headache comorbidities, patient treatment preferences and response to current care [15, 67, 68]. While more research is needed to understand this finding, one possible explanation is that engagement with headache diagnosis leads to more practitioner certainty about their own capacity to provide sufficient preventative management for those with primary headaches. With increasing examination of the quality and integration of health services and providers engaged in preventative headache management [20, 69], more research examining the factors that influence headache patient co-management between chiropractors and other headache providers is warranted.

Our findings identified chiropractors engaged in primary headache diagnosis are more likely to refer headache patients to investigate a headache red-flag. This finding is not unexpected, since the use of headache diagnostic criteria is more likely to result in the identification of headache features associated with headache red-flag findings. The most important diagnostic consideration for frontline clinicians engaged in headache management is to rule out headaches caused by serious and potentially life-threatening underlying pathology. While rare, the underlying causes associated with headache red-flag symptoms can include stroke, sub-arachnoid haemorrhage, tumour, meningitis and artery dissection (carotid or vertebral) [70]. Since headache features in those with an underlying brain tumour can be similar to those of tension headache and migraine [71], and since neck stiffness and headache in those with underlying meningitis and arterial dissection [72, 73], can be similar to those with cervicogenic headache, chiropractors need to be mindful of the possibility of serious underling pathology when examining those who present with headache.

Our study found that chiropractors engaged in primary headache diagnosis are more likely to use soft tissue therapies such as massage, myofascial technique, stretching or trigger-points to neck/shoulder area for their patients with tension headache. This finding is interesting given a recent systematic review which found manual therapies, including soft tissue therapies, may be more effective than pharmacological care for reducing the short-term frequency, intensity and duration of tension headache [58]. Tenderness of myofascial trigger points of the neck and shoulder muscles are increased in patients with tension-type headache [74, 75]. These active trigger-points appear to cause nociceptive input that contributes to peripheral and central sensitization in patients with chronic tension headache [76]. While further research is needed, these findings appear to support soft tissue treatment approaches that are specifically aimed at addressing these muscular factors.

## Limitations

Regression analysis of chiropractors engaged in primary headache diagnosis provides an excellent opportunity to better understand the primary headache management associated with this common headache provider. The self-reported nature of the data collected is a limitation of our study – the data may be subject to recall bias and the use of Likert categories are subject to practitioner interpretation. The headache management characteristics of chiropractors reported in this study may also be influenced by non-respondents to the survey when estimating chiropractors who use of ICHD primary headache criteria and the associations related to their headache management characteristics. Nonetheless, analysis of this cross-sectional survey provides valuable insights into primary headache health management associated with these popular providers and helps to identify key questions for further enquiry into chiropractic headache management.

## Conclusion

Our research found that most chiropractors managing primary headaches are engaged in primary headache diagnosis and that this practice is likely to influence their clinical-decision making toward key aspects of primary headache patient management and co-management. These findings highlight the need for closer examination of the clinical decision-making that underlies chiropractic primary headache management and the role of these providers toward reducing the burden of this significant public health issue. Gathering this information will help to improve our understanding of the role of chiropractors within multimodal, multidisciplinary headache patient management.

## Abbreviations

ACORN: Australian Chiropractic Research Network
CAM: Complementary and alternative medicine
HDI: Headache Disability Inventory
ICHD: International Classification of Headache Disorders
MIDAS: Migraine disability assessment questionnaire
PBRN: Practice-based research network
